# Nutrient Enrichment Increases Blue Carbon Potential of Subtropical Seagrass Beds

**DOI:** 10.1111/gcb.70401

**Published:** 2025-08-02

**Authors:** Bridget F. Shayka, Sean Richards, Mona A. Andskog, Jacob E. Allgeier

**Affiliations:** ^1^ Department of Ecology and Evolutionary Biology University of Michigan Ann Arbor Michigan USA; ^2^ Faculty of Science and Engineering Southern Cross University Lismore New South Wales Australia

**Keywords:** biomass allocation, carbon burial, carbon turnover, climate change, long‐term experiment, nature‐based solutions, nitrogen, nutrient supply rate, phosphorus, production

## Abstract

Seagrass beds have potential as nature‐based solutions to climate change because their high rates of primary production can bury large amounts of carbon. Yet, realizing their potential necessitates improved understanding of the mechanisms contributing to carbon burial, especially in the context of nutrient enrichment, a ubiquitous threat to seagrass beds globally. Leveraging a nine‐year nutrient enrichment experiment, we tested how different nutrient sources, supply rates, and ratios altered mechanisms underpinning carbon burial. Nutrient enrichment increased aboveground and decreased belowground biomass but increased carbon production and turnover, particularly belowground. To inform conservation efforts, we showed that blade height and shoot density effectively predict belowground carbon turnover and therefore provide a simple measure to assess potential belowground carbon inputs to sediment. By identifying the mechanisms that promote carbon burial in the face of nutrient enrichment, our study advances understanding of how to prioritize protection of nature‐based solutions amidst this ubiquitous stressor.

## Graphical abstract

Seagrass beds have tremendous potential to mitigate climate change because their high rates of primary production bury large amounts of carbon. They are also globally threatened by nutrient pollution, and thus, realizing this potential requires understanding how this stressor affects carbon burial. We leveraged a nine‐year experiment to test how nutrient enrichment alters carbon burial in seagrass. Contrary to expectations, we found that nutrient enrichment increased carbon production and turnover, particularly in belowground biomass and thus should increase carbon burial. Our findings highlight that even seagrass ecosystems experiencing moderate enrichment should be prioritized for protection for their carbon burial potential.

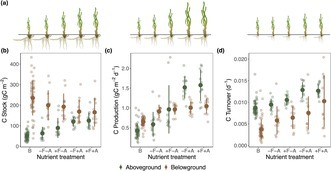

## 1 Introduction

Nature‐based solutions (NBSs) that contribute to the removal of carbon (C) from the atmosphere represent a critical component of the portfolio of solutions necessary for combating climate change. A prerequisite of NBSs is not only that they remove C from the atmosphere but that this removed carbon is stored in stocks that are inactive on geological scales (James et al. 2024). Ecosystems with high rates of primary production are often the focus of efforts to understand and implement NBSs to combat climate change (James et al. 2024), likely because higher primary production can lead to potentially more C available for long‐term storage (Arnaud et al. 2023; Duarte et al. 2010; Larkum et al. 2006). Yet, because most of the burial of C occurs through belowground ecosystem stocks (Keller et al. 2022; Larkum et al. 2006; Macreadie et al. 2014; Zou et al. 2021), it is necessary to understand the mechanisms that govern belowground production and biomass turnover. However, these belowground processes are often poorly understood, in part due to how difficult they are to study, particularly in coastal ecosystems despite their vast potential to serve as NBSs.

**FIGURE 1:**
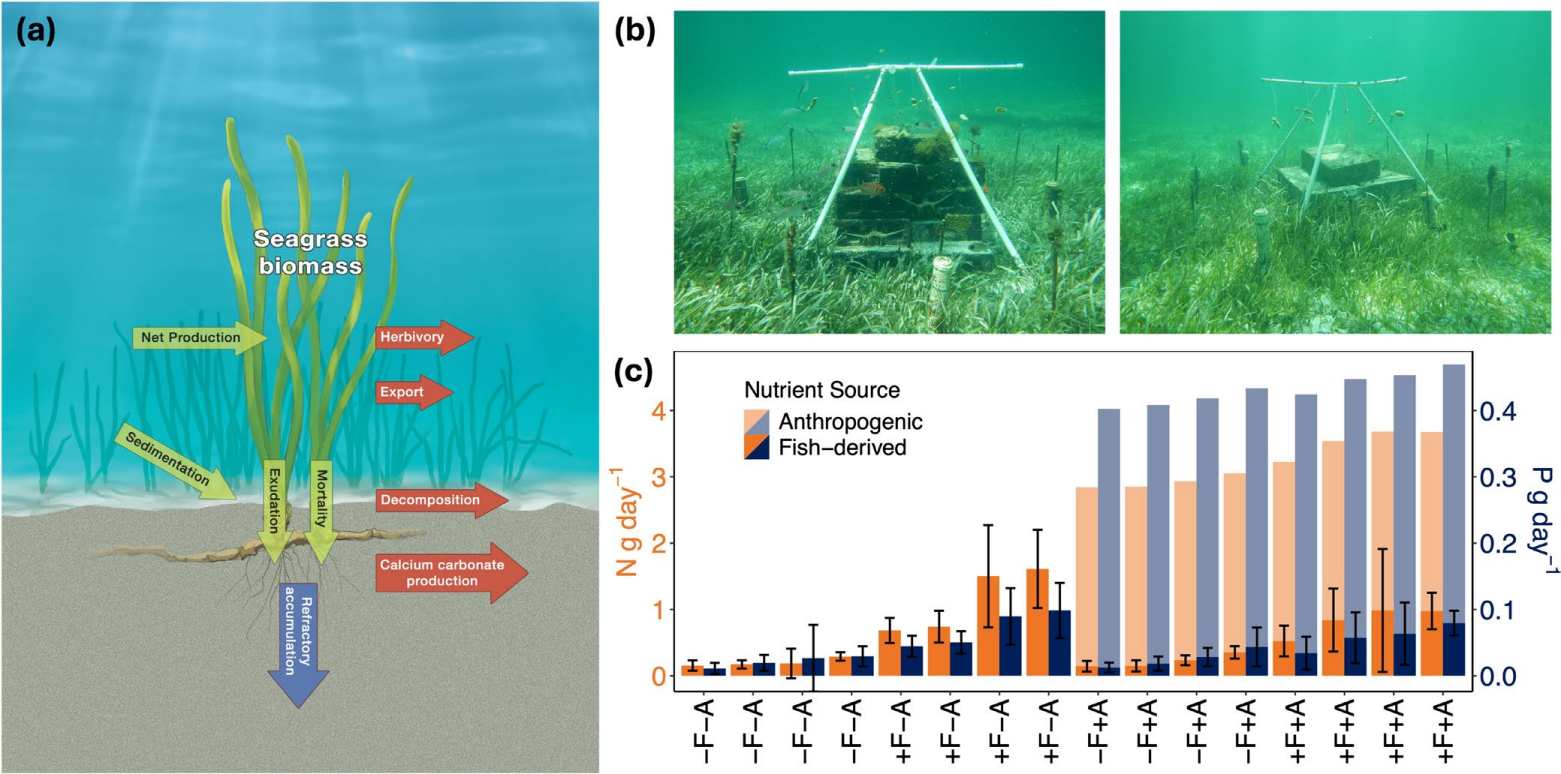
(a) Conceptual diagram of the fluxes involved in carbon burial in seagrass ecosystems. Seagrass biomass carbon stock is at the top and sediment carbon stock is at the bottom. Green arrows indicate inputs, red arrows indicate outputs. Refractory accumulation leads to long‐term carbon burial. Illustration by John Megahan. (b) Photos of artificial reefs used to alter nutrient supply to seagrass, both with anthropogenic nutrient addition via white PVC diffusers near ground. Left photo has high fish biomass (+F + A) and right photo has low fish biomass (−F + A). Credit: Jacob Allgeier. (c) Nutrient supply rates at each reef. *X*‐axis labels represent treatments: −F = lower fish‐derived nutrients, +F = higher fish‐derived nutrients, −A = without anthropogenic nutrients, +A = with anthropogenic nutrients. N = nitrogen, P = phosphorus. Error bars on fish‐derived nutrients are standard deviation.

Seagrass ecosystems are commonly cited NBSs for climate change due to their high production and substantial C storage (Duarte et al. 2010; James et al. 2024; Lima et al. 2023; Mcleod et al. 2011; Rifai et al. 2023; Shayka et al. 2023). It is now well established that seagrass beds contain large C stocks in their sediment and biomass (Fourqurean et al. 2012; Shayka et al. 2023), but research on their role as net sinks or sources of C is still evolving (Howard et al. 2018; Macreadie et al. 2017; Unsworth et al. 2022; Van Dam et al. 2021). Fully understanding the processes by which C burial in seagrass occurs requires measuring all C fluxes including both organic and inorganic components in the system (Figure 1a), some of which are still poorly understood (Howard et al. 2018; Macreadie et al. 2017; Van Dam et al. 2021). The most important pathways are those that result in the accumulation of refractory forms of C that are most resistant to decomposition and can be retained in the system for long timescales (decades to millennia) (Larkum et al. 2006). While these pathways are highly complex, autochthonous C inputs are ultimately driven by the production of new biomass (Figure 1a) (Larkum et al. 2006). Notably, belowground production represents a large fraction of total seagrass production (including the blades aboveground, and the roots and rhizomes belowground; Figure 1a) (Duarte et al. 1998; Esquivel et al. 2022), and is also a primary driver of the amount of C that is shunted into the sediments as refractory C that has low decomposition rates (Duarte et al. 2013; Larkum et al. 2006). Yet, to more effectively relate production to C burial, the rate at which the biomass of the belowground tissue turns over, that is, the ratio of production (g m^−2^ time^−1^) to biomass (g m^−2^) – herein biomass turnover, is a particularly useful metric that must be better understood because it effectively characterizes inputs of C from belowground biomass to the sediment (Gill and Jackson 2000; Keller et al. 2022).

Seagrass ecosystems are among the most impacted by human activities globally (Waycott et al. 2009). Of the many stressors to these ecosystems, anthropogenic nutrient enrichment and overfishing are among the most pervasive (Burkholder et al. 2007; Howarth 2008). Importantly, they both modify nutrient inputs to seagrass ecosystems by increasing and decreasing, respectively, the supply rate of critical nutrients, namely nitrogen (N) and phosphorus (P), and the ratio by which they are available for seagrass uptake (Allgeier et al. 2013, 2015, 2018; Fourqurean and Zieman 2002; Howarth 2008). Because of the ubiquity of altered nutrient dynamics in seagrass ecosystems globally, the importance of nutrients for aboveground seagrass production (Allgeier et al. 2018; Esquivel et al. 2022; Ferdie and Fourqurean 2004; Qin et al. 2021b), biomass allocation (Armitage et al. 2011; Layman et al. 2016), and C storage (Armitage and Fourqurean 2016; Qin et al. 2021a) is relatively well understood. In contrast, there is a scarcity of research assessing the effects of nutrients on seagrass belowground production and biomass turnover, greatly inhibiting our understanding of the mechanisms that underpin C burial in these ecosystems and how anthropogenic impacts are altering them. For example, understanding if the supply of individual nutrients, for example, N or P, versus their ratio best predicts primary production has long been a focus of ecological theory (Elser et al. 2007; Redfield 1958; Tilman 1982; van der Ploeg et al. 1999), and is also highly relevant for management because it helps identify how best to regulate nutrient inputs to ecosystems. Yet, to date, our understanding of the importance of supply rate versus ratio is limited to aboveground production and storage in seagrasses (Allgeier et al. 2018). Effective implementation of seagrass beds as NBSs to climate change necessitates improved understanding of the impacts of nutrient enrichment on the processes, both above‐ and belowground, that underpin their ability to bury C and identification of simplified predictors of C burial.

We used a nine‐year long‐term nutrient enrichment experiment and field manipulation to test: (1) how a gradient of nutrient supply rates and ratios from anthropogenic and fish‐derived nutrients affected the storage of nutrients and allocation of biomass above‐ and belowground in seagrass, and (2) how changes in storage across this gradient relate to changes in above‐ and belowground biomass production. We also identified the best static measure of seagrass for estimating C biomass turnover to address the conservation need for easily measurable seagrass traits to serve as proxies for potential C inputs to sediment. To do this, we used artificial reefs constructed in subtropical seagrass beds in The Bahamas as replicate experimental units to manipulate fish biomass to create a gradient of fish‐derived nutrient supply to mimic overfishing in conjunction with anthropogenic nutrient enrichment. Specifically, we answered three questions:

Q1: How do supply rates and ratios of nutrients from different sources affect biomass, C, and nutrient storage above‐ and belowground, and aboveground production in seagrass ecosystems?

Q2: How do different sources and rates of nutrient enrichment alter the underlying causal relationships that drive aboveground seagrass production?

Q3: How are C stock, C production, and C turnover in above‐ and belowground seagrass biomass affected by different sources and rates of nutrient enrichment?

## 2 Materials and Methods

### 2.1 Experimental Design

This study was conducted in the Bight of Old Robinson, Abaco, The Bahamas—a shallow (~3 m deep) semi‐enclosed bay with a mosaic of seagrass meadows dominated by *Thalassia testudinum* . Sixteen 30‐cinder block artificial reefs were constructed (December 2010) as experimental units for testing hypotheses of nutrient enrichment on seagrass (Figure 1b) (see Allgeier et al. 2018). Ambient water column nutrients in this system are very low. For example, water column samples taken ~1 m from all experimental reefs resulted in low values of soluble reactive phosphorus (3.7 ± 3.8 μg/L), total phosphorus (4.9 ± 2.2 μg/L), nitrate + nitrite (3.2 ± 3.6 μg/L), and total nitrogen (109.4 ± 27.1 μg/L). Importantly, these values are similar to ambient water column nutrients collected within the same embayment and similar systems nearby (~3.5 μg/L soluble reactive phosphorus, ~3 μg/L nitrate, and ~7 μg/L ammonium; Allgeier et al. 2010; Stoner et al. 2011). These findings highlight the extent to which ambient water column nutrient concentrations are low, and they demonstrate no apparent increase in water column nutrients from the enrichment by the fish or fertilizer—likely because uptake rates by the producers in the system are extremely high and increase with greater primary production (Lee and Dunton 1999). Collectively, these findings are consistent with previous research conducted by the authors in this system (Allgeier et al. 2011, 2018, BFS and JEA personal observation) and those from nearby seagrass beds in Florida (Fourqurean and Zieman 2002). Further, ambient nutrient concentrations do not necessarily reflect nutrient supply to the system because they are influenced by both input to and uptake in the system. For all of these reasons, nutrient supply rates were used in analyses instead of water column nutrient concentrations.

To understand how seagrass responded to gradients in supply rates and ratios, we took advantage of a long‐term experimental reef study that sought to understand the effects of different nutrient sources on aboveground seagrass production and diversity (Allgeier et al. 2018). Specifically, nutrient supply to seagrass surrounding the reefs was altered in two ways: (1) simulated anthropogenic nutrient enrichment via the addition of fertilizer, (2) altered fish‐derived nutrient supply to mimic overfishing via manipulation of the reef structure (Figure S1a). These two treatments were imposed on the artificial reefs in a 2 × 2 factorial design: with and without anthropogenic nutrients crossed with high and low fish‐derived nutrients. The experiment was a block design (*n* = 4 blocks), each block consisting of the four treatment types.

Anthropogenic nutrient enrichment was simulated using PVC diffusers filled with 500 g of slow‐release fertilizer (Florikan 18–6–8 NPK; N is 9.7:8.3 ammonium: nitrate). Seven diffusers were suspended ~0.5 m above the substrate and evenly placed in a circle at a distance of ~0.75 m from each reef. Diffusers were changed approximately every 3 months for 9 years. Nutrient release rates from diffusers were calculated based on the percentage loss of fertilizer from a subset of diffusers (*n* = 7) after deployment (2.7 ± 0.3 SD, 0.39 ± 0.042 SD g reef^−1^ d^−1^, for N and P, respectively; molar N:P = 15.3; Allgeier et al. 2018).

Fish‐derived nutrient supply was altered by reducing the physical complexity on the reefs by filling in the holes of the cinder blocks, thereby substantially altering fish biomass and community composition (Allgeier et al. 2018). Because the exact density of fish could not be directly manipulated, the high/low fish treatment also provided a continuous gradient of fish‐derived nutrient supply across all 16 reefs, ranging from 0.14 to 1.61 g N reef^−1^ d^−1^ and 0.01 to 0.10 g P reef^−1^ d^−1^ (N:P of fish supply ranged from 15.6 to 37.0) (Figure 1c). N:P of total nutrient supply to all reefs from anthropogenic and/or fish‐derived nutrients ranged from 15.4 to 37.0. Fish‐derived nutrient supply was quantified by modeling species‐specific nutrient supply rates onto repeated visual census data that estimated fish abundance and size. This approach has been conducted previously by the authors on these same reefs (Allgeier et al. 2018, 2020), in adjacent reefs within the same embayment (Allgeier et al. 2013), and in other work (Allgeier et al. 2014, 2015, 2016) (see Appendix S1: Supplemental Methods for further detail). We used an average value of N and P nutrient supply from all repeated surveys over the course of enrichment for each reef (*n* = ~10 surveys per reef over the 9 years of enrichment).

### 2.2 Seagrass Responses

Samples for this study were collected on all reefs after 9 years of enrichment, providing a measure of the long‐term effects of enrichment on seagrass parameters. Specifically, 24 seagrass responses (aboveground production, shoot density, epiphyte biomass, bite count, as well as C content, N content, P content, C isotopes, and biomass of blades, sheaths, rhizomes, and roots) were measured to determine the impacts of the nutrient treatments on seagrass production and C dynamics. Responses were measured at 1 and 20 m from each reef along three transects radiating from different sides of the reef (Figure S1b). The responses at 20 m from the reefs represented a control treatment reflective of background nutrient conditions because the effects of reef nutrient treatments are not seen that far from the reefs (Allgeier et al. 2018; Layman et al. 2013, 2016).

To calculate seagrass aboveground production, three areas (10 × 10 cm) were demarcated with stakes at each distance perpendicular to each transect (herein called core areas because this is where we also took cores at depth for belowground biomass, *n* = 288, Figure S1b). Holes were poked in all the *T. testudinum* shoots within each core area (*n* = 2978 across all) to measure growth via the standard hole‐punch method (Zieman 1974). After 11–12 days, all seagrass within the core area was removed to 14 cm deep using flat blades and shovels. Growth rates were measured on all shoots with visible puncture holes (up to 100% of the shoots in a core) and averaged within each core area. The average growth rate for each core was multiplied by the total number of shoots in each core (including those without growth measurements) to determine total blade growth rate per core (g day^−1^) and then multiplied by 100 to calculate the aboveground production per square meter (g m^−2^ day^−1^).

Collected seagrass from each core was separated by species, with *T. testudinum* being dominant in all cores. ( *Syringodium filiforme* , the only other seagrass species present, was less than 15% of the biomass in 95% of all cores, in the remaining it was less than 40%.) After *T. testudinum* growth rates were measured and shoots were counted, its biomass was separated by plant part—blades, sheaths, rhizomes, and roots. Blades were rinsed with DI water and scraped of epiphytes using a razor blade, and fish bites (semicircle notches in blades) were counted on each blade (*n* = 8430). Epiphytes from each shoot were lyophilized together for 24 h and then weighed. Blades and a subsample of sheaths, rhizomes, and roots from each core were thoroughly cleaned of sand with DI water, lyophilized for 24 h, and weighed. These subsamples were then ground with a ball mill grinder (Precellys Evolution) and used for determination of nutrient content of each plant part in each core – %C, %N, δ^13^C (Carlo Erba NC2500 [Italy]), and %P (acid digestion and colorimetric analysis via spectrophotometer [Fourqurean and Zieman 2002]). The remaining biomass of each part was briefly dipped in 10% HCl to remove fine grain sand that was otherwise difficult to remove by hand, rinsed in DI water, lyophilized for 24 h, and weighed. This weight was combined with the subsample weight to quantify total biomass of each plant part in each core. Values of all responses were averaged between the three cores (subsamples) at each distance on each transect.

### 2.3 Statistical Analyses

#### 2.3.1 Question 1

The goal was to understand how well each of the 24 response variables was predicted by the supply rate or ratio of nutrients (continuous predictors) and the sources of nutrients—fish‐derived or anthropogenic (categorical predictors). To do this, we conducted a series of linear models with categorical predictors and continuous predictors. For these analyses, we only used data from seagrass sampled from 1 m for each reef because at this distance we were confident in the amount of nutrients from fishes or fertilizer the seagrass was exposed to. To simplify comparisons among the different models, responses were grouped into three broad categories: quantity (production, biomass, shoot density, and seagrass C isotopes—which is often used as a proxy for C production), quality (seagrass nutrient and C content, and epiphyte biomass—higher biomass indicates higher quality of food resource for consumer), and herbivory (bite count).

- To compare the effects of the two nutrient sources, we used linear mixed effects models with anthropogenic and fish‐derived nutrient enrichment as separate categorical predictors with two levels each and block as a random effect (response = anthropogenic + fish‐derived + (anthropogenic × fish‐derived) + block). Responses were transformed to meet model assumptions of normality and homoscedasticity if necessary and standardized for comparison of effect sizes between responses. For each model, we determined which predictor was significant and the direction and magnitude of each effect.
- To determine the effects of continuous supply of N, P, and their ratio (N:P) across the 16 reefs on each seagrass response, we used three separate mixed effects linear models with block as a random effect. Each model contained either N supply, P supply, or N:P supply (quantified from anthropogenic and fish‐derived nutrients) as a continuous predictor (all standardized). Because those values are often correlated, separate models were used for comparison. We compared the effect sizes of each nutrient for each response and AIC values between models to determine the best‐fit model (ΔAIC < 2). Sheath %C and root %C models did not meet assumptions and were left out of all Q1 analyses.

#### 2.3.2 Question 2

To understand how the source and supply rate of nutrient enrichment affected the causal relationships that drive seagrass aboveground production, we used structural equation models (SEMs) on three subsets of the data that were previously determined to be useful for comparison—fish‐derived + anthropogenic nutrients (treatments with anthropogenic nutrients, including both high and low fish), fish‐derived nutrients only (treatments with no anthropogenic nutrients, including both high and low fish), and background nutrient control (20 m) – herein nutrient “scenarios” (Allgeier et al. 2020; Andskog et al. 2023). Each SEM included the following parameters: aboveground production, epiphyte biomass, bite count, shoot count, and biomass, %C, %N, %P, and δ^13^C for each plant part (blades, sheaths, rhizomes, roots). For each scenario, we first tested hypothesized relationships between all predictors (see Figure S3 for model and rationale) and then iterated through relationships to find the best‐fit model using the *piecewiseSEM* package in R v4.4.0 (Grace et al. 2012; Lefcheck 2016). Because we were most interested in understanding the causal relationships that predicted aboveground production, we limited our final interpretation to the primary and secondary predictors (i.e., direct and indirect effects) of production in the best‐fit SEM models.

#### 2.3.3 Question 3

To understand how nutrient enrichment affected seagrass C biomass turnover in aboveground and belowground compartments, we conducted three analyses. First, belowground production was calculated by incorporating our values of aboveground biomass, aboveground production, and belowground biomass that were measured as explained in *Seagrass Responses* into an existing seagrass ecosystem model (Esquivel et al. 2022; see further details of belowground production calculation in Appendix S1: Supplemental Methods). Belowground C production was then quantified from these estimates by multiplying belowground production by belowground %C—calculated by summing rhizome %C and root %C weighted based on rhizome and root biomass proportions. Aboveground C production was calculated by multiplying aboveground production by blade %C (both measured empirically). Total C production was calculated by summing the aboveground and belowground C production values, and C biomass turnover was calculated by dividing C production of each biomass group (aboveground, belowground, and total) by its respective C stock.

Second, to understand the impacts of the different nutrient scenarios on C stock, C production, and C turnover in aboveground, belowground, and total biomass (nine total response variables) we used individual linear mixed effects models that included nutrient treatment as a categorical predictor with five levels and block as a random effect.

Third, to determine whether C biomass turnover could be predicted via simplistic observational measures, we tested the efficacy of two easy‐to‐measure seagrass traits for predicting C biomass turnover. We ran individual linear models for aboveground, belowground, and total C biomass turnover with blade height and shoot density as predictors in each model (C turnover = blade height + shoot density).

## 3 Results

### 3.1 Question 1: How Do Supply Rates and Ratios of Nutrients From Different Sources Affect Biomass, C, and Nutrient Storage Above‐ and Belowground, and Aboveground Production in Seagrass Ecosystems?

**FIGURE 2:**
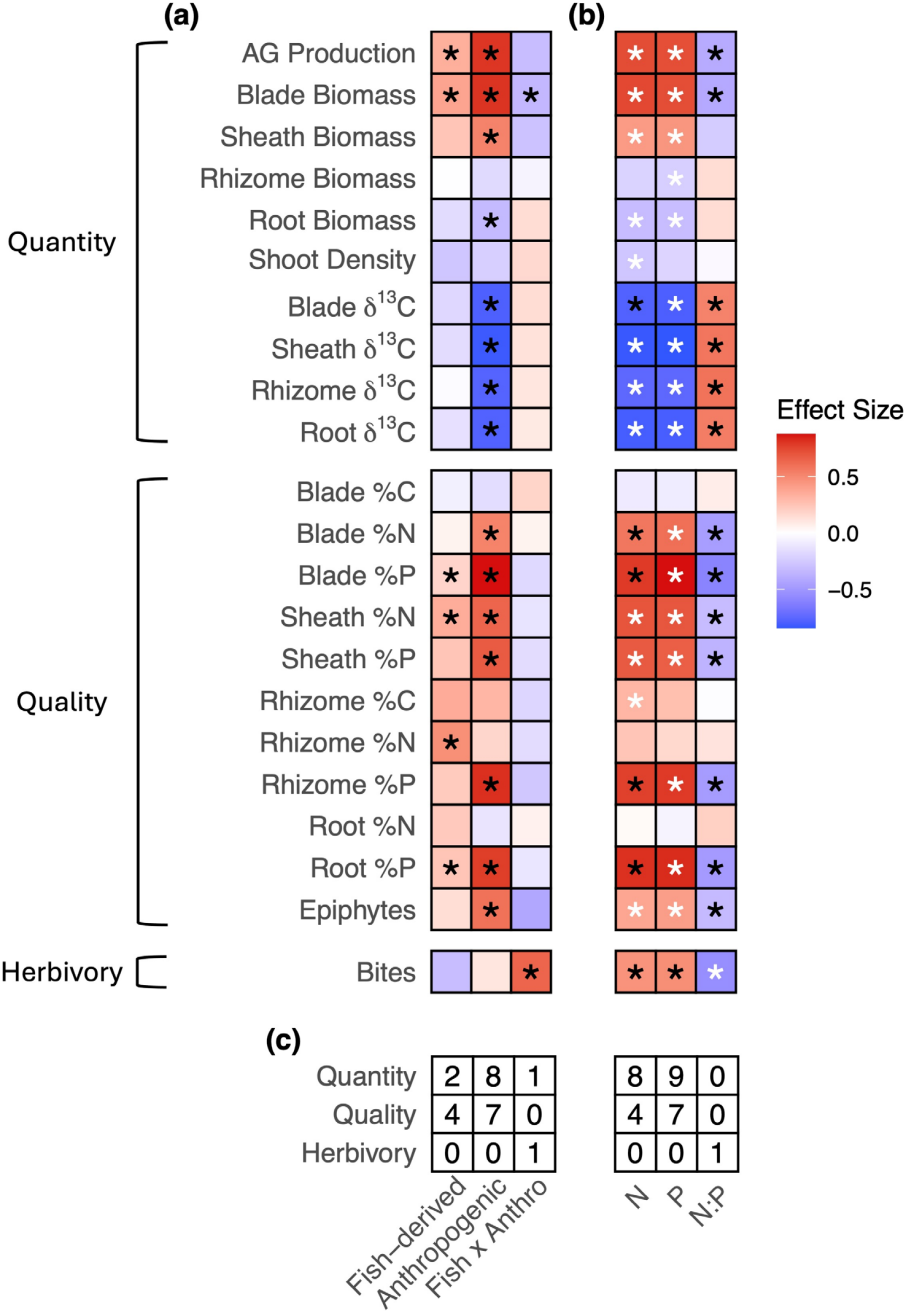
(a) Impacts of different sources of nutrients on the suite of seagrass responses. Each row is an individual model. Effect size indicates standardized effect of increasing fish‐derived or anthropogenic nutrients or of the interaction between the two factors on each seagrass response. (b) Impacts of N, P, and N:P supply on the suite of seagrass responses. Each cell is an individual model. Effect size indicates standardized effect of increasing N, P, or N:P ratio on each seagrass response. Red colors are positive effects, and blue colors are negative effects. Predictors with an asterisk were statically significant (*p*‐value < 0.05). White asterisks in (b) indicate best models for each response variable (ΔAIC < 2). If two models were within 2 ΔAIC, both asterisks are white. (c) Summary of number of responses in each group best explained by each predictor variable.

Nutrient enrichment via anthropogenic and fish‐derived nutrients, both from N and P, had a positive effect on aboveground biomass and production and a negative effect on belowground biomass (Figure 2, Figure S2). Anthropogenic nutrient enrichment significantly affected more seagrass responses than fish‐derived nutrients. Interestingly, the effects of fish‐derived and anthropogenic nutrients rarely interacted, suggesting that nutrients from either source typically had similar effects on seagrass attributes despite the larger quantity of anthropogenic nutrients (Figure 2). P better predicted the responses of seagrass quality than N or N:P, and N and P were approximately equally good at predicting responses of seagrass quantity (Figure 2c). Herbivory was the only response for which N:P was a better predictor than N or P, despite having a significant effect on many responses.

### 3.2 Question 2: How Do Different Sources and Rates of Nutrient Enrichment Alter the Underlying Causal Relationships That Drive Aboveground Seagrass Production?

**FIGURE 3:**
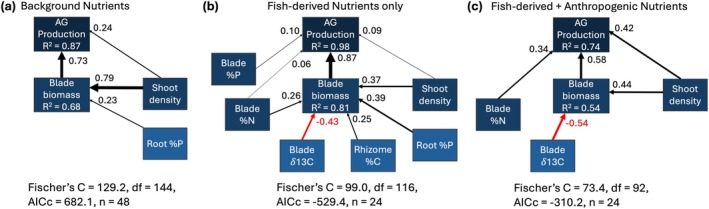
Best‐fit SEMs for predictors of AG production for each nutrient scenario—simplified to only include primary and secondary predictors of AG production. Arrow width is proportional to the standardized effect size, given next to the arrows. Black arrows indicate positive effects; red arrows indicate negative effects. Shade of blue lightens with distance from AG production.

In all cases, SEM models built from our hypothesized causal relationships between different seagrass attributes and aboveground production fit the data poorly (*p*‐value < 0.05; note *p*‐value < 0.05 for an SEM indicates that the modeled relationships were significantly different from those suggested by the data) (Figure S3). The best‐fit SEMs contained 19 significant relationships in both the background nutrient scenario and the scenario with only fish‐derived nutrients, and contained 17 relationships in the scenario with fish‐derived and anthropogenic nutrients (Figure S4, Table S1). Of those relationships, very few represented primary or secondary predictors of aboveground production—four under background nutrients, nine with fish‐derived nutrient addition, and five with fish‐derived and anthropogenic nutrients (Figure 3).

When considering only the primary and secondary predictors, a clear pattern emerged whereby the number of predictors needed to explain variation in aboveground production was greatest under the fish‐mediated nutrient scenario and fewest under background conditions. In all three nutrient scenarios, blade biomass and shoot density positively affected aboveground production, with blade biomass having the strongest effect (Figure 3). Blade %N had an increasingly large positive effect on production as nutrient supply increased (Figure 3). Conversely, root %P had a positive effect on blade biomass under background nutrient conditions and with fish only, but this relationship was not retained in the model with anthropogenic nutrient enrichment (Figure 3). The strength of the negative effect of blade δ^13^C on blade biomass increased with nutrient addition (Figure 3), reflecting increased usage of the light isotope (increased fractionation) under nutrient enrichment.

### 3.3 Question 3: How Are C Stock, C Production, and C Turnover in Above‐ and Belowground Seagrass Biomass Affected by Different Sources and Rates of Nutrient Enrichment?

**FIGURE 4:**
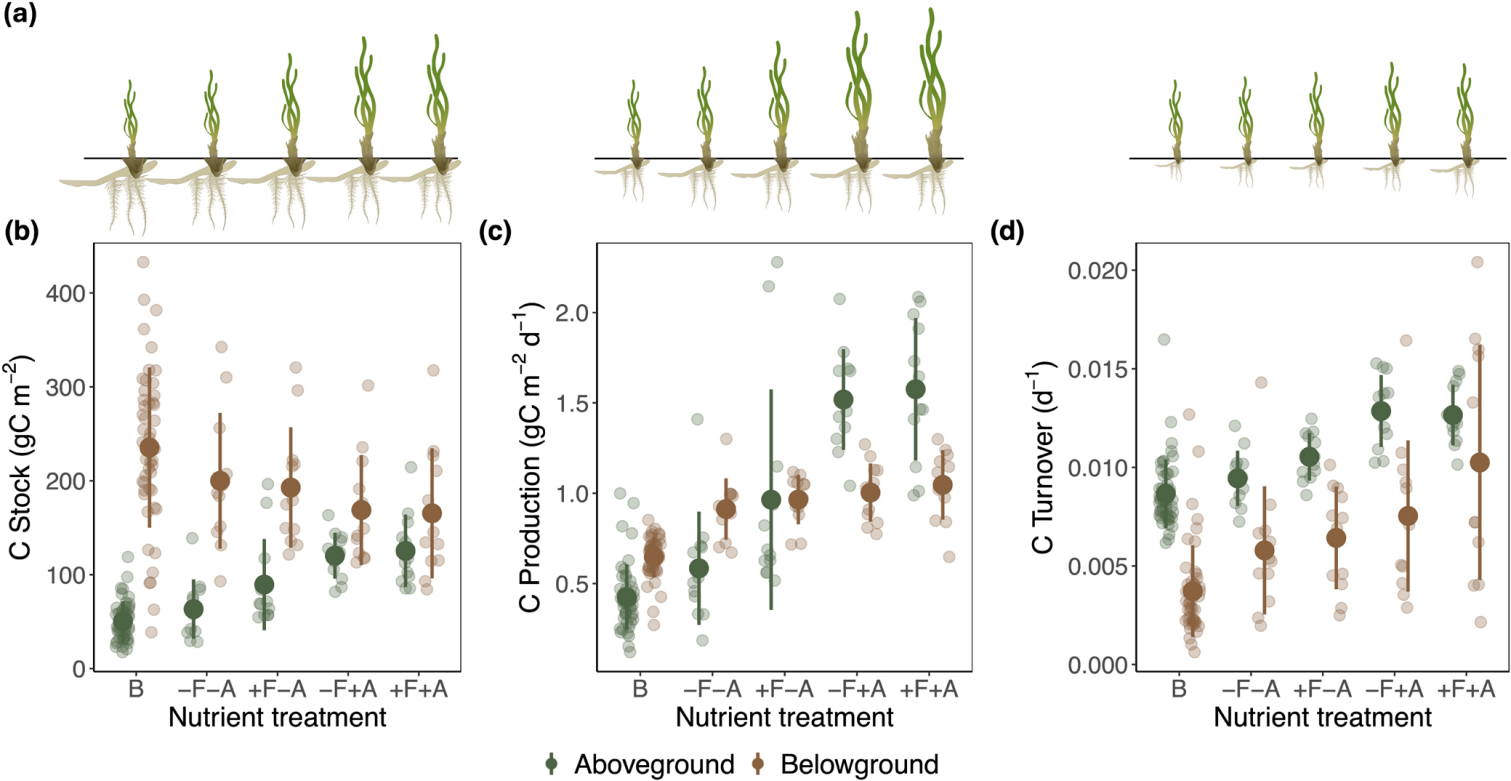
(a) Seagrass images sized in relative proportions of mean aboveground and belowground C stock, C production, and C biomass turnover for seagrass in each nutrient treatment. (b–d) C stock, C production, and C turnover in aboveground and belowground seagrass biomass. Solid point is mean, and error bars are standard deviations. Labels represent treatments: B = background nutrients, −F = lower fish‐derived nutrients, +F = higher fish‐derived nutrients, −A = without anthropogenic nutrients, +A = with anthropogenic nutrients.

In all cases, C production and C turnover in biomass (aboveground, belowground, and total) increased with greater nutrient enrichment (Figure 4, Figure S5). Aboveground C stock increased while belowground C stock decreased with greater nutrient enrichment (Figure 4, Figure S5), likely due to changes in biomass as opposed to changes in %C (Figure 2).

**FIGURE 5:**
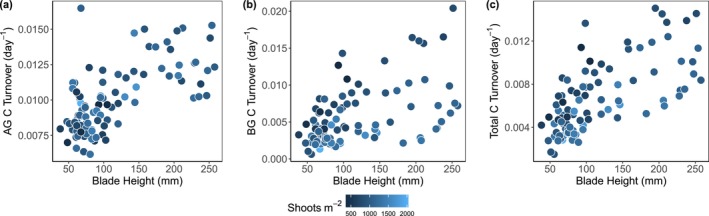
Correlation plots of aboveground (AG), belowground (BG), and total carbon (C) turnover with blade height and shoot density. Adjusted *R* ^2^ of models: AG C turnover = 0.41, BG C turnover = 0.49, total C turnover = 0.62.

Blade height and shoot density, two of the easier seagrass traits to measure in the field, were significant predictors of C turnover. Blade height was a significant positive predictor for aboveground, belowground, and total C turnover (Figure 5). Shoot density was a significant negative predictor of belowground and total C turnover (Figure 5). Overall, the models explained a substantial proportion of the variation in the data (adj *R* ^2^: AG = 0.41, BG = 0.49, total C turnover = 0.62). Correlation plots of other seagrass traits with respect to C turnover are included in the Supplements for comparison (Figures S6–S8).

## 4 Discussion

Seagrass ecosystems have the potential to serve as highly valuable nature‐based solutions (NBSs) to climate change due to their high rates of production. In particular, conservation of areas with high carbon burial potential could maximize the role of seagrass in mitigating climate change via the sequestration and storage of carbon dioxide from the atmosphere. However, to take advantage of the capacity of these ecosystems to mitigate climate change and to effectively manage them, it is necessary to understand the factors that best predict production and C biomass turnover under real‐world conditions that may affect these processes directly, for example, nutrient enrichment. In our study, we leveraged a nine‐year nutrient enrichment experiment to quantify the predictors of seagrass production under various nutrient enrichment scenarios. Our findings advance existing ecological theory on nutrient enrichment and inform seagrass conservation as a NBS for climate change in three ways: (1) We found that nutrient enrichment increased aboveground biomass and decreased belowground biomass, shifted aboveground production from P limitation to N limitation, and increased C production and C biomass turnover. (2) We found that nutrient supply rate was a stronger driver of seagrass responses than nutrient source or supply ratio, contrary to expectations of the resource ratio hypothesis and ecological stoichiometry. (3) We showed that two easily measurable seagrass traits, blade height and shoot density, can be used as proxies for belowground C biomass turnover and therefore can be used by conservationists and managers to assess the potential of a given ecosystem as a NBS. Overall, these findings contribute to an improved mechanistic understanding of the ability of seagrass to respond to nutrient pollution, a ubiquitous stressor, and serve as a NBS to climate change.

A strength of our experimental design is the ability to test for effects of two different nutrient sources as well as the supply rate and ratio of two different nutrients over the course of 9 years. We found that the effect sizes of most responses were greater in magnitude with anthropogenic nutrient enrichment compared to fish‐derived nutrients. This is likely in part due to the fact that our experimental enrichment supplied anthropogenic nutrients at a rate that was on average slightly higher than that supplied by fishes (Figure 1c). This suggests that nutrient supply rate is a stronger driver of seagrass responses than source. Yet, it is important to note that seagrass has a higher affinity for ammonium (Cornelisen and Thomas 2004) than other forms of N (e.g., nitrate, nitrite). This suggests that because fish release N mostly as ammonium (Anderson 2001) and fertilizer typically includes nitrate (Broschat and Moore 2007; fertilizer used in our study was 9.7:8.3 ammonium:nitrate), in some cases fish‐derived nutrients may have stronger effects than anthropogenic nutrients—which is not what we found in our study.

That nutrient supply rate is likely a leading driver of changes in seagrass attributes is also supported by our findings that the supply rate, in contrast to the ratio, was always, save one example (herbivory), the best predictor of all seagrass responses. This finding is contrary to resource ratio theory and ecological stoichiometry that state that nutrient ratios should be important predictors of primary production (Sterner and Elser 2002; Tilman 1982; Tilman et al. 1982). Our findings are, however, consistent with phytoplankton research that demonstrated supply rates can be more important than the nutrient ratio for growth in some species (Lagus et al. 2004; Nhu et al. 2019) and with previous research on these reefs focused on seagrass community structure and function (Allgeier et al. 2018). Notably, the N:P effect was generally opposite of the effects of N and P for a given response (i.e., if N and P had a positive effect, N:P had a negative effect on a given response) because the fertilizer used in this experiment had a low N:P ratio and thus, higher nutrient supply correlated with lower N:P. This correlation suggests that the negative effect of N:P on bite count is due to greater herbivory in treatments with higher nutrient supply and in which the quality of plant tissues is greater due to higher nutrient content (and likely epiphyte loads). Importantly, herbivory from smaller grazers (not megafauna such as turtles [Gulick et al. 2021]) has been shown to not have strong effects on aboveground biomass in this system (Allgeier et al. 2018; Andskog et al. 2023; Brines et al. 2022; data herein) and thus is unlikely to have strong effects on the ability of the system to sequester and store C. These findings from 9 years of nutrient enrichment, in conjunction with past research (Allgeier et al. 2018), are consequential for conservation efforts in tropical seagrass beds because they identify that the absolute not the relative amount of nutrients should be a management priority.

If seagrass production responds greater to supply rates than ratios, identifying which nutrient has the strongest effect is an important challenge. Our structural equation models (SEMs) clearly showed that seagrass is highly plastic with respect to which nutrient most limits production. For example, under very low levels of nutrient availability (background), production was P‐limited—a finding commonly supported in tropical seagrass ecosystems (Allgeier et al. 2018; Andskog et al. 2023; Brines et al. 2022; Koch et al. 2001; Smith 1984). As nutrient availability increased under conditions of fish‐derived nutrient supply, production became colimited—demonstrating that even under modest supply of P (Figure 1c—fish excretion in this system is typically N rich; Allgeier et al. 2013, 2015), P‐limitation can at least in part be alleviated. Finally, under conditions of both anthropogenic and fish‐derived nutrients, production shifted entirely to N‐limitation—likely due to the low N:P ratio of the fertilizer (Figure 1c). These findings highlight two key things: (1) seagrass production is sensitive to the availability of different nutrients, (2) seagrass can still be highly productive under variable nutrient conditions (Figure 4). The implications of these findings for conservation are significant—primary production in oligotrophic seagrass beds is tolerant of variable nutrient environments, which underscores their great potential as NBSs in the impacted coastal environments of the Anthropocene.

Excessive nutrient enrichment in seagrass ecosystems is typically associated with reduced production and biomass, often due to light limitation from algal blooms (Lee et al. 2007; Lefcheck et al. 2018; McGlathery et al. 2007). Among the most important findings of our study, and one that further underscores the utility of seagrass as a NBS, was that in a well‐flushed embayment, even relatively high levels of enrichment resulted in greater C production and C biomass turnover, both above‐ and belowground. This finding is important because the higher belowground C production and C biomass turnover should lead to higher C exudation or mortality in belowground biomass (Kengdo et al. 2023), increasing C inputs from seagrass to the sediment. Yet how this affects C burial per se, we were unable to measure. For example, we found that nutrient enrichment resulted in increased aboveground and decreased belowground C stocks (Figure 4), likely due to a shift in resource allocation (Esquivel et al. 2022; Layman et al. 2016). Further, it is unknown how belowground C production and C biomass turnover of the other seagrass species in the experimental plots, *S. filiforme* , differs from *T. testudinum* . Another important concern is that increased nutrient availability can also lead to increased microbial activity and decomposition in seagrass ecosystems, potentially offsetting C inputs from biomass turnover via increased respiration that exports C into the water column (Qin et al. 2021a; Zhang et al. 2022). Thus, while our findings provide important new insight into the mechanisms that drive seagrass ecosystem C burial, further research incorporating inorganic with organic C dynamics is needed, particularly with respect to sediment responses to water column enrichment.

Notably, our study experimentally quantifies C production and C biomass turnover in a seagrass system that is not otherwise experiencing excessive eutrophication. High nutrient enrichment can lead to seagrass loss due to shading by epiphytes, macroalgae, or phytoplankton, which would result in the loss of stored C (Lee et al. 2007; Lefcheck et al. 2018; McGlathery et al. 2007; Schmidt et al. 2012). Because anthropogenic nutrient inputs are widespread on coastlines globally (Burkholder et al. 2007; Howarth 2008; Malone and Newton 2020), management attention must be focused on reducing these inputs to minimize seagrass loss. However, because our findings indicate that seagrass is somewhat resistant to enrichment, they suggest that in nutrient‐limited systems, nutrient inputs may not need to be reduced to pristine levels to have a positive impact on the system and maximize C fixation. This is an important finding because completely removing all nutrient enrichment from human sources is nearly impossible. Nonetheless, there is an imperative for some level of nutrient management for seagrass ecosystem health because if enrichment is high enough to increase water column nutrients, seagrass can be negatively impacted by light limitation from phytoplankton production (McGlathery et al. 2007). Note that while our enrichment experiment supplied nutrients at realistically high levels, we found no evidence of light limitation, which other research efforts in this same system indicate is unlikely because it is relatively well‐flushed (Shayka and Allgeier 2025). Another valuable takeaway for conservation from our study may be that minimizing overfishing could increase the productivity and potentially C burial of seagrass systems and thus may be an overlooked pathway to increase the potential of these ecosystems to serve as NBSs.

One of the greatest factors inhibiting the potential of coastal ecosystems to serve as NBSs is our relative inability to easily quantify the important ecological processes that underpin C burial. A key contribution of our study was to clearly demonstrate that two of the easier measurements to conduct in the field, seagrass blade height and shoot density, are able to predict C biomass turnover with impressive levels of accuracy. We acknowledge that although these aboveground measurements are easier to conduct than belowground measurements, they still require nontrivial effort to quantify at useful spatial and temporal scales. However, current efforts to quantify these traits via areal imagery (Schill et al. 2021) strongly suggest that these traits will be able to be estimated via remote surveillance in the future. We also acknowledge that there is error in these models, and they do not account for other C fluxes in the system. The underlying mechanisms driving these responses should be investigated further to understand, for example, how tradeoffs between blade height and shoot density relate to intraspecific competition, and thus impact production and turnover above and belowground. Despite these caveats, these traits together explained a large amount of the variation in total C biomass turnover, which means that they can be used directly by conservationists and managers to understand the relative potential of different ecosystems for C biomass turnover and potentially burial. To further aid managers in using these metrics, it would be beneficial to ground truth these findings in other regions with other species assemblages. By combining fundamental contributions to seagrass C budgets with practical tools for estimating C biomass turnover in seagrass beds, our findings improve our ability to assess the capacity of seagrass ecosystems to take up and store carbon and therefore to serve as NBSs to climate change under continued anthropogenic nutrient enrichment.

## Author Contributions

**Bridget F. Shayka:** conceptualization, data curation, formal analysis, methodology, visualization, writing – original draft, writing – review and editing. **Sean Richards:** formal analysis, writing – review and editing. **Mona A. Andskog:** data curation, methodology, writing – review and editing. **Jacob E. Allgeier:** conceptualization, funding acquisition, methodology, resources, supervision, visualization, writing – original draft, writing – review and editing.

## Conflicts of Interest

The authors declare no conflicts of interest.

## Supporting information


**Appendix S1:** gcb70401‐sup‐0001‐Supinfo.pdf.

## Data Availability

The data and code that support the findings of this study are openly available in Zenodo at https://doi.org/10.5281/zenodo.14796993.
