# Supplementary material for: Nutrient Enrichment Increases Blue Carbon Potential of Subtropical Seagrass Beds
Source: Glob Chang Biol. 2025 Aug 2;31(8):e70401. doi: 10.1111/gcb.70401 (PMC12317336; doi:10.1111/gcb.70401)
Supplement: Supplementary file 1 — Appendix S1: gcb70401‐sup‐0001‐Supinfo.pdf. [file GCB-31-e70401-s001.pdf]

Supporting Information

Supplemental Methods

Fish-derived nutrient supply calculations

Fish abundance was estimated using underwater visual census. Surveys were conducted every three years, multiple times per year on each reef. All surveys were conducted during the day by the same observer (J.E.A). At each survey time, all fishes on a reef were identified to the species level, and size (total length) of each individual was estimated to the nearest cm. Survey duration was as long as required to record all fishes present on the reef, typically <10 minutes. Previous work on similar reefs in this system has shown that fish community assembly plateaus at ~120 days after reef construction (Yeager et al., 2011), and all of our surveys were conducted after this time period. Previous work on the same reefs used in our study showed no temporal difference in fish communities on the same reef in terms of total N supply, P supply, and biomass at five timepoints over the course of a year (Allgeier et al., 2018). Average fish biomass per reef ranged from 162 g on a low-fish reef to 4113 g on a high-fish reef.

Fish nutrient supply rates and ratios for N and P were estimated using models generated by Allgeier et al. (2014, 2015) and the census data. Estimates were summed across all individuals within a reef at each census date and then averaged across dates by reef to yield mean supply rates and ratios per reef over time following Allgeier et al. (2013). Averaging across dates provides a more reliable approximate of fish nutrient supply, and is less biased by variation that can be induced with a snapshot fish census (Allgeier et al., 2013; Layman et al., 2013; Yeager et al., 2011). We did not account for nutrient input from egestion (feces) from fishes in our

estimates for three reasons: (1) Egestion is not immediately available to primary producers because it is organic waste (unlike excretion) and thus only becomes available for producers after it has been remineralized by microbes. (2) The amount of nutrients in egestion is small relative to excreted nutrients from metabolic waste (Schreck & Moyle, 1990), so we do not expect that this impacted our findings substantially. (3) While we can generate these values from our energetic models, it is difficult to quantify empirically, and a key strength of our models is the integration of robust empirical data on excretion with energetics models. Average fish-derived N and P supplies at each reef are shown in Figure 1a.

Model for belowground (BG) production calculations

The goal of the model was to estimate BG production by calibrating the model to mirror patterns found in the empirical data. Specifically, relationships between AG vs BG biomass and AG biomass vs AG production were used to calibrate model output with empirical findings. Because empirical relationships were different between 1m and 20m data, we used separate models for 1m and 20m distances.

A well-established seagrass model rooted in rich *in-situ* observations was parameterized using empirical data from this study including AG and BG minimum and maximum biomass. The model was adapted from an individual-based model developed by Esquivel et al. (2022) that included a base ecosystem model of a seagrass ecosystem. The model was underpinned by a single-nutrient (N) production model that simulated seagrass (*T. testudinum*) growth with individual fish that swam around the model environment (DeAngelis, 1992). Here, we only used the base seagrass ecosystem model, which was 50mx50m and included a 1 m^2 artificial reef in

the center. For the purposes of this analysis and following Esquivel et al. (2022), the seagrass ecosystem was a closed system.

In the model, nutrients were assimilated from the water column and differentially allocated to aboveground (AG) and belowground (BG) seagrass biomass in response to nutrient availability based on empirical findings from this system (Layman et al. (2016) and data herein). Each 1 m^2 cell in the model contained three nutrient pools: (1) the water-column nutrients, (2) aboveground and belowground seagrass biomass and (3) detrital biomass. Nutrients cycle through these pools through three processes: (1-2) nutrient uptake, allocation, and production; (2-3) seagrass biomass slough (i.e. loss); and (3-1) detrital mineralization.

The model was simulated for 10 years. Nutrients were added to the system every 120 minutes (hereafter referred to as 1 timestep) (see next paragraph). Seagrass growth was simulated every 12 timesteps (once a day) according to nutrients available in the cell. Separate model scenarios were run to reflect 1m and 20m environmental conditions to best approximate the empirical patterns.

To understand how production changed as a function of nutrient enrichment, we stochastically increased the nutrient loading to each cell incrementally over the course of the model run (Fig S9). We incorporated variation in the nutrient loading rates as they increased with time to simulate the variability in nutrient supply from fishes and fertilizer across our experiment gradient. Daily loading rates ranged from 0.0257 g N m^{-2} day^{-1} to 2.756 g N m^{-2} day^{-1} for 1 m (mean = 1.138 g N m^{-2} day^{-1}) and 0.00642 g N m^{-2} day^{-1} to 0.698 g N m^{-2} day^{-1} for 20 m (mean = 0.287 g N m^{-2} day^{-1}). The amount of nutrient enrichment was based on the relative range of nutrients supplied by fishes, approximated enrichment from our experiment, and reflected the range of enrichment found in our experiment (Allgeier et al. (2014, 2018), data herein).

To generate empirical relationships from the model, we systematically varied model parameters to align model data with two empirical relationships for both 1m and 20m seagrass conditions: (***1***) AG biomass and AG production; (***2***) AG biomass and BG biomass (Fig S10). (***1***) To align AG biomass and AG production, we varied: (***i***) the amount of nutrients supplied (modified scalar) and (***ii***) the proportion of seagrass biomass sloughed (Fig S10, AG Biomass/Prod Regressions). (***2***) To align AG biomass and BG biomass, we varied nutrient allocation to aboveground production (seagrass allocation function) (Fig S10, AG/BG Regressions). These parameters were varied for 1m and 20m separately such that model data closely approximated empirical relationships (Fig S10).

To predict the BG Biomass and BG production relationship, we fitted generalized additive models (GAMs) to the model's BG biomass-production relationships for 1m and 20m and predicted BG production across the range of empirical data (Fig S11).

References

Allgeier, J. E., Layman, C. A., Montana, C. G., Hensel, E., Appaldo, R., & Rosemond, A. D. (2018). Anthropogenic versus fish-derived nutrient effects on seagrass community structure and function. *Ecology*, *99*(8), 1792–1801. https://doi.org/10.1002/ecy.2388

Allgeier, J. E., Layman, C. A., Mumby, P. J., & Rosemond, A. D. (2014). Consistent nutrient storage and supply mediated by diverse fish communities in coral reef ecosystems. *Global Change Biology*, *20*(8), 2459–2472. https://doi.org/10.1111/gcb.12566

Allgeier, J. E., Layman, C. A., Mumby, P. J., & Rosemond, A. D. (2015). Biogeochemical implications of biodiversity and community structure across multiple coastal ecosystems. *Ecological Monographs*, *85*(1), 117–132. https://doi.org/10.1890/14-0331.1

Allgeier, J. E., Yeager, L. A., & Layman, C. A. (2013). Consumers regulate nutrient limitation regimes and primary production in seagrass ecosystems. *Ecology*, *94*(2), 9. https://doi.org/10.1890/12-1122.1

DeAngelis, D. L. (1992). *Dynamics of Nutrient Cycling and Food Webs*. Springer Dordrecht. https://doi.org/10.1007/978-94-011-2342-6

Esquivel, K. E., Hesselbarth, M. H. K., & Allgeier, J. E. (2022). Mechanistic support for increased primary production around artificial reefs. *Ecological Applications*, *32*(6), e2617. https://doi.org/10.1002/eap.2617

Layman, C. A., Allgeier, J. E., & Montaña, C. G. (2016). Mechanistic evidence of enhanced production on artificial reefs: A case study in a Bahamian seagrass ecosystem. *Ecological Engineering*, *95*, 574–579. https://doi.org/10.1016/j.ecoleng.2016.06.109

Layman, C. A., Allgeier, J. E., Yeager, L. A., & Stoner, E. W. (2013). Thresholds of ecosystem response to nutrient enrichment from fish aggregations. *Ecology*, *94*(2), 530–536. https://doi.org/10.1890/12-0705.1

Schreck, C. B., & Moyle, P. B. (Eds.). (1990). *Methods for fish biology*. American Fisheries Society.

Yeager, L. A., Layman, C. A., & Allgeier, J. E. (2011). Effects of habitat heterogeneity at multiple spatial scales on fish community assembly. *Oecologia*, *167*(1), 157–168. https://doi.org/10.1007/s00442-011-1959-3

Supplemental Table

Table S1. Table of full SEM results of best models for each nutrient scenario. Values are standardized effect sizes. F + A = fish-derived + anthropogenic nutrients, F only = fish-derived nutrients only, Background = background nutrients. Green and Red shading indicate the direction of the relationship, positive and negative, respectively.

Response	Predictor	F + A	F only	Background
AG Production	Blades	0.5764	0.8705	0.7344
AG Production	Shoots	0.4248	0.0894	0.2372
AG Production	Blade N	0.3353	0.0644	
AG Production	Blade P		0.1045	
Blades	Shoots	0.4443	0.3711	0.7894
Blades	Blade d13C	-0.5413	-0.4291	
Blades	Blade N		0.2622	
Blades	Rhizome C		0.2514	
Blades	Root P		0.3883	0.2333
Sheaths	AG Production	0.5684		0.8383
Sheaths	Blades		0.6768	
Sheaths	Shoots		0.4095	
Sheaths	Sheath C	-0.3749		
Sheaths	Sheath N			-0.2951
Rhizomes	Sheaths	0.4399	0.8375	
Rhizomes	Shoots	0.3794		0.4907
Rhizomes	Blade C		0.3651	
Rhizomes	Blade P			0.5055
Rhizomes	Sheath N		-0.3583	
Rhizomes	Rhizome d13C	0.375		0.318
Rhizomes	Rhizome P			-0.4831
Rhizomes	Epiphytes			-0.3654
Rhizomes	Bites			0.2532
Roots	Rhizomes	0.8368	0.9313	0.6986
Roots	Blades	-0.3671		
Roots	Sheaths			0.2077
Roots	Blade d13C	-0.3409		
Roots	Blade C	-0.184		

Roots	Blade N			0.1671
Roots	Root d13C	0.2432		-0.2608
Roots	Root N	-0.2647		
Epiphytes	Blade d13C		0.612	
Epiphytes	Blade N		-0.4389	
Epiphytes	Blade P		0.7407	
Epiphytes	Sheath P			0.3022
Epiphytes	Root C			-0.444
Bites	Sheath N			-0.3359
Bites	Rhizome C		-0.4393	
Bites	Root N	0.6704		

Supplemental Figures

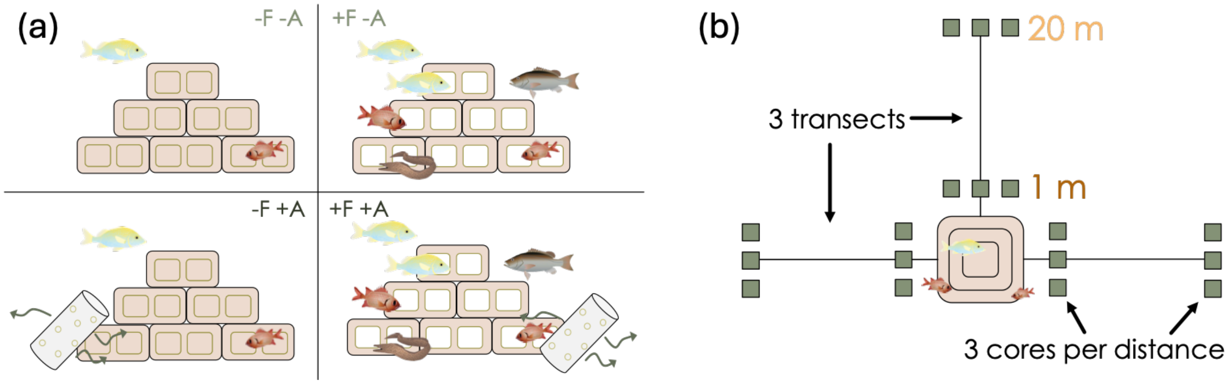

Figure S1. (a) Illustration of the four nutrient treatment types. -F = lower fish-derived nutrients, +F = higher fish-derived nutrients, -A = without anthropogenic nutrients, +A = with anthropogenic nutrients. (b) Illustration of the core sampling regime along transects at each reef.

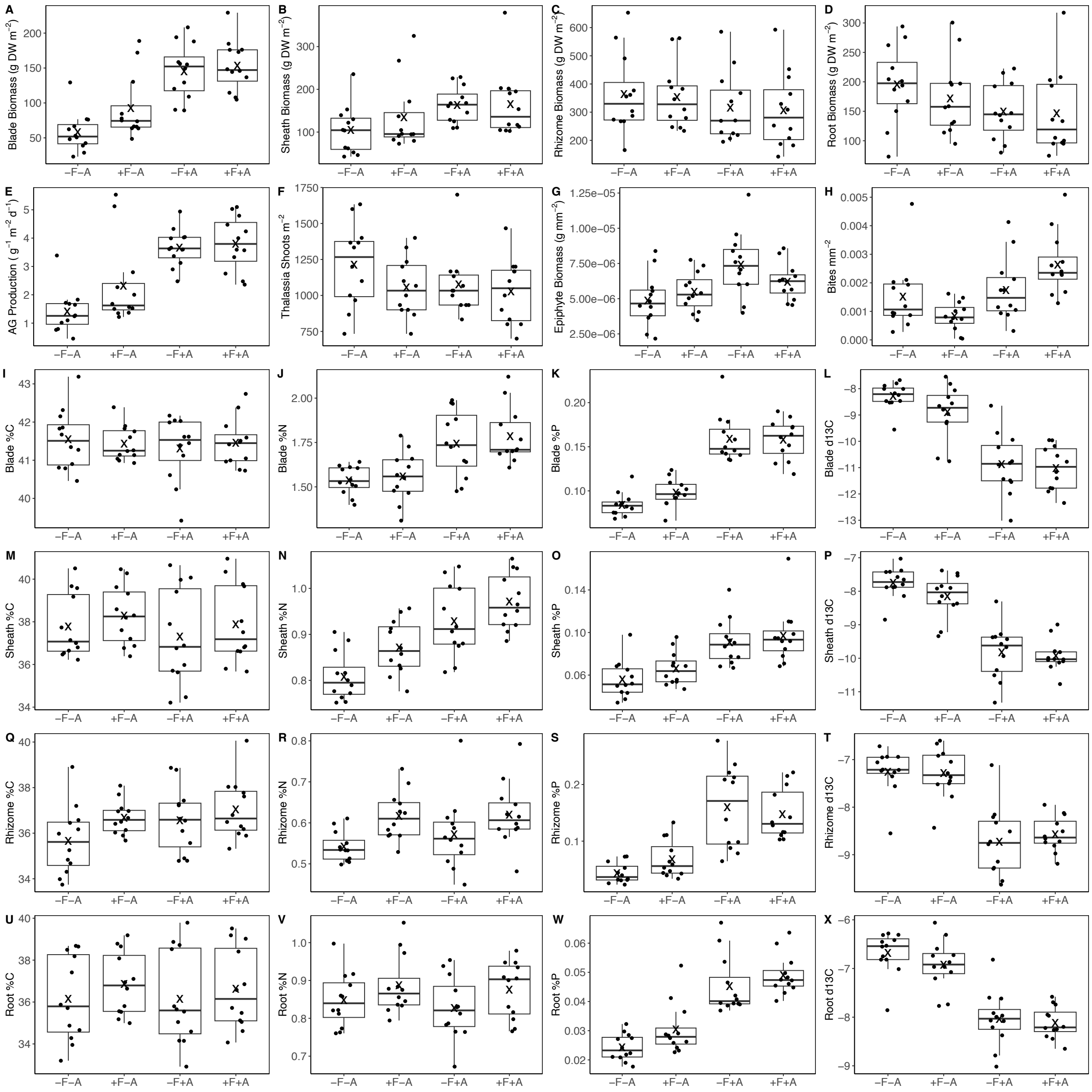

Figure S2. Data underlying the results represented in Fig 2a. X-axis labels represent treatments: -F = lower fish-derived nutrients, +F = higher fish-derived nutrients, -A = without anthropogenic nutrients, +A = with anthropogenic nutrients. Each plot is a different response variable. All responses are not standardized or transformed for visualization on plots. X symbol on boxplots is the mean.

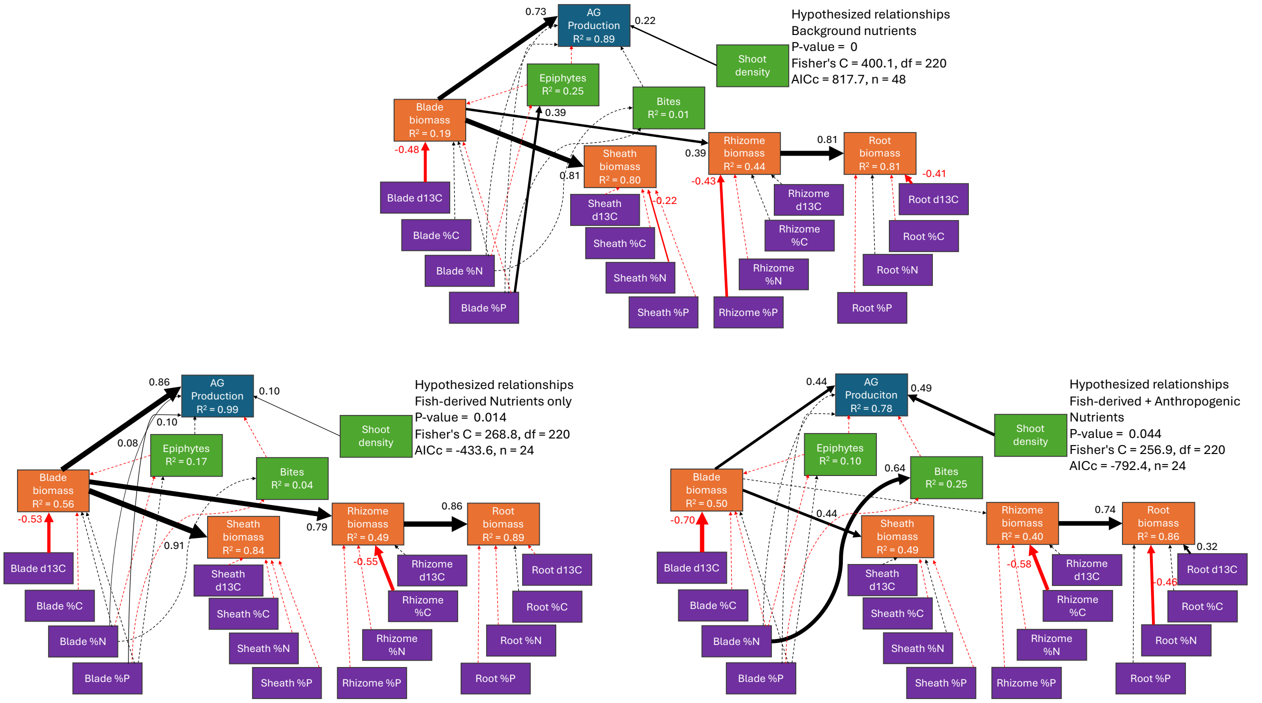

Figure S3. Tested hypothesis SEM for each nutrient scenario. For all scenarios, we predicted that AG (aboveground) production would be directly predicted by blade biomass, blade %N, blade %P, epiphyte biomass, bite count, and shoot density. We predicted that $\delta^{13}C$, %C, %N, and %P of each plant part (blades, sheaths, rhizomes, and roots) would predict the biomass of the respective plant part. We predicted that blade biomass would predict sheath and rhizome biomass, and that rhizome biomass would predict root biomass. Although we did not hypothesize that sheath, rhizome, and root biomass would affect AG production, we included them and their hypothesized predictors in the model because the drivers of seagrass AG production are not well understood. We wanted to leave open the possibility that there is a path between these variables and AG production that is currently unidentified. We confirmed that the sample size was adequate to run all models.

P-value < 0.05 indicates that the model is significantly different from the relationships predicted by the data (i.e. some relationships need to be added or removed for the model to accurately

reflect the data). Sold lines indicate significant paths; dashed lines indicate non-significant paths. Arrow width is proportional to the standardized effect size, given next to the arrows. Black arrows indicate positive effects; red arrows indicate negative effects.

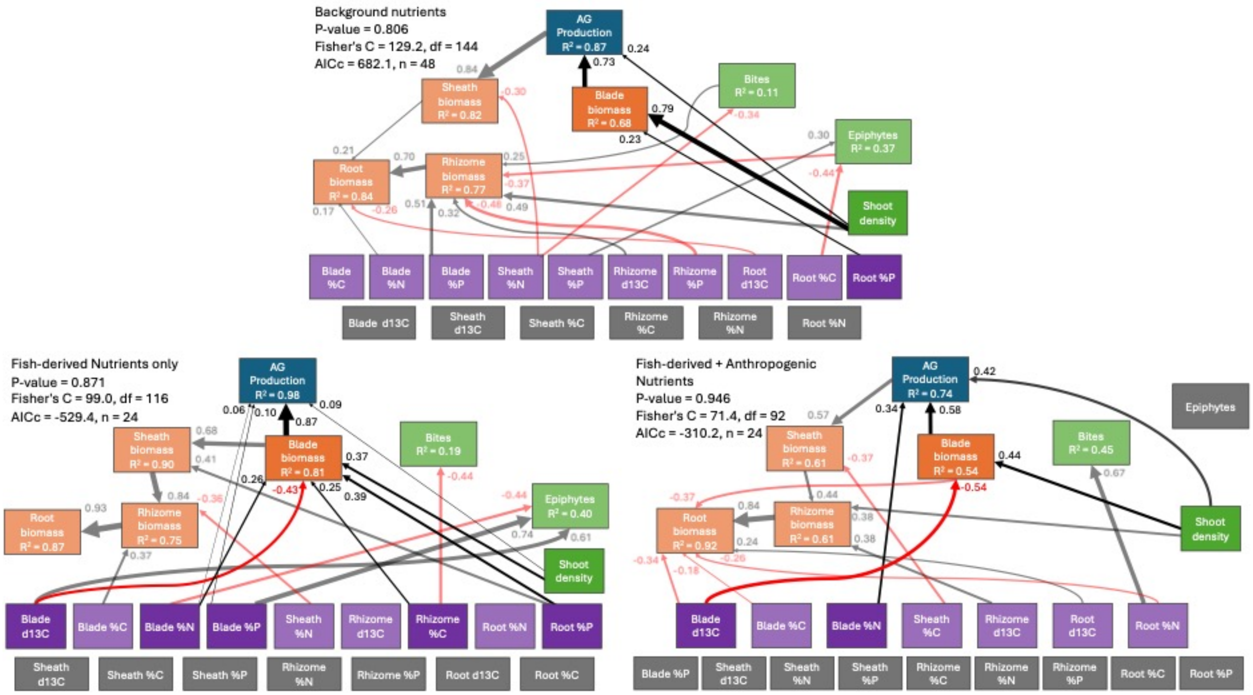

Figure S4. Full best-fit SEM for each nutrient scenario. P-value > 0.05 indicates that the model is not significantly different from the relationships predicted by the data. Primary and secondary drivers of aboveground production have more saturated colors. Arrow width is proportional to the standardized effect size, given next to the arrows. Black arrows indicate positive effects; red arrows indicate negative effects.

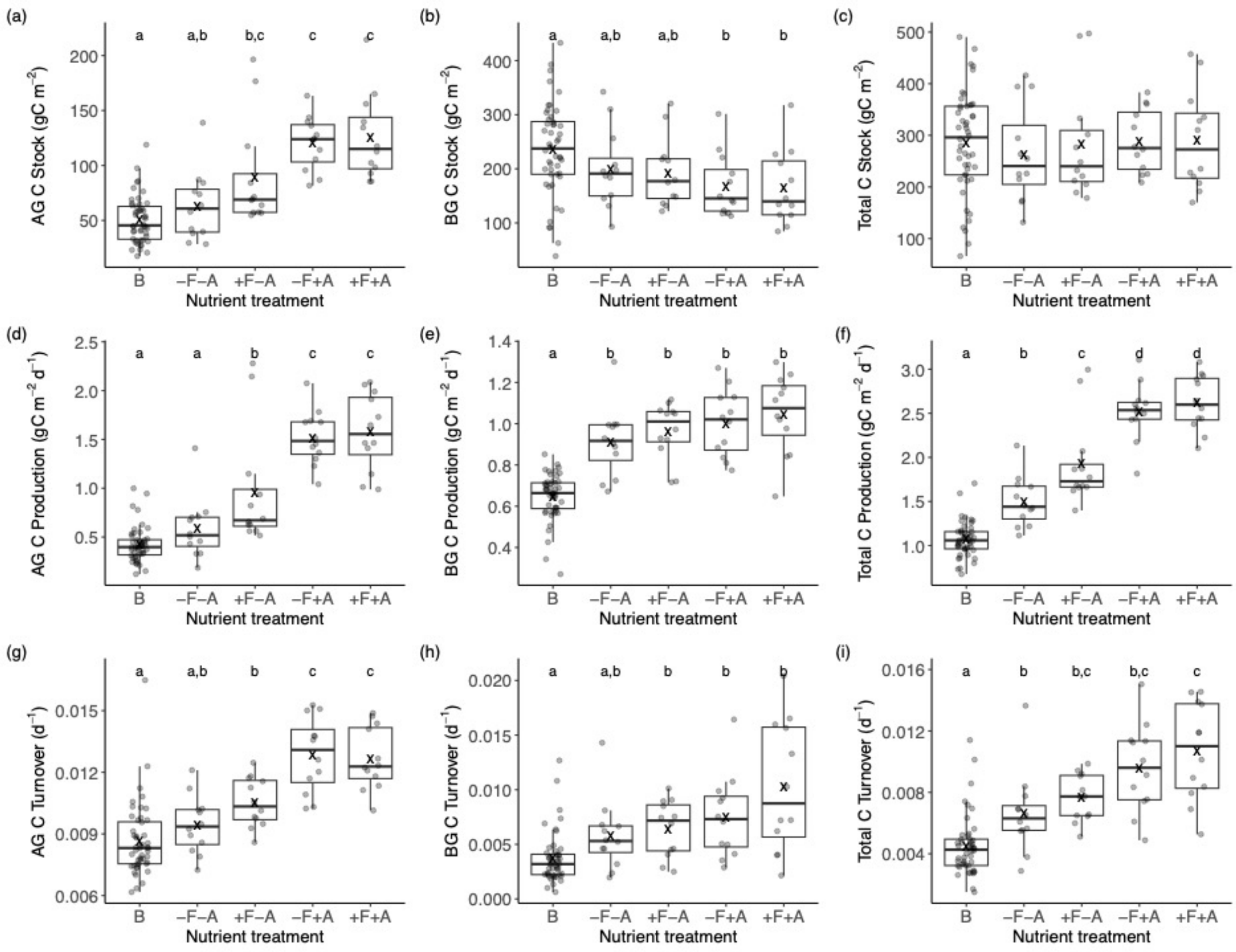

Figure S5. AG C = aboveground carbon, BG C = belowground carbon. X-axis labels represent treatments: B = background nutrients, -F = lower fish-derived nutrients, +F = higher fish-derived nutrients, -A = without anthropogenic nutrients, +A = with anthropogenic nutrients. X symbol on boxplots is the mean. Letters above data represent significant differences via Tukey HSD post-hoc tests.

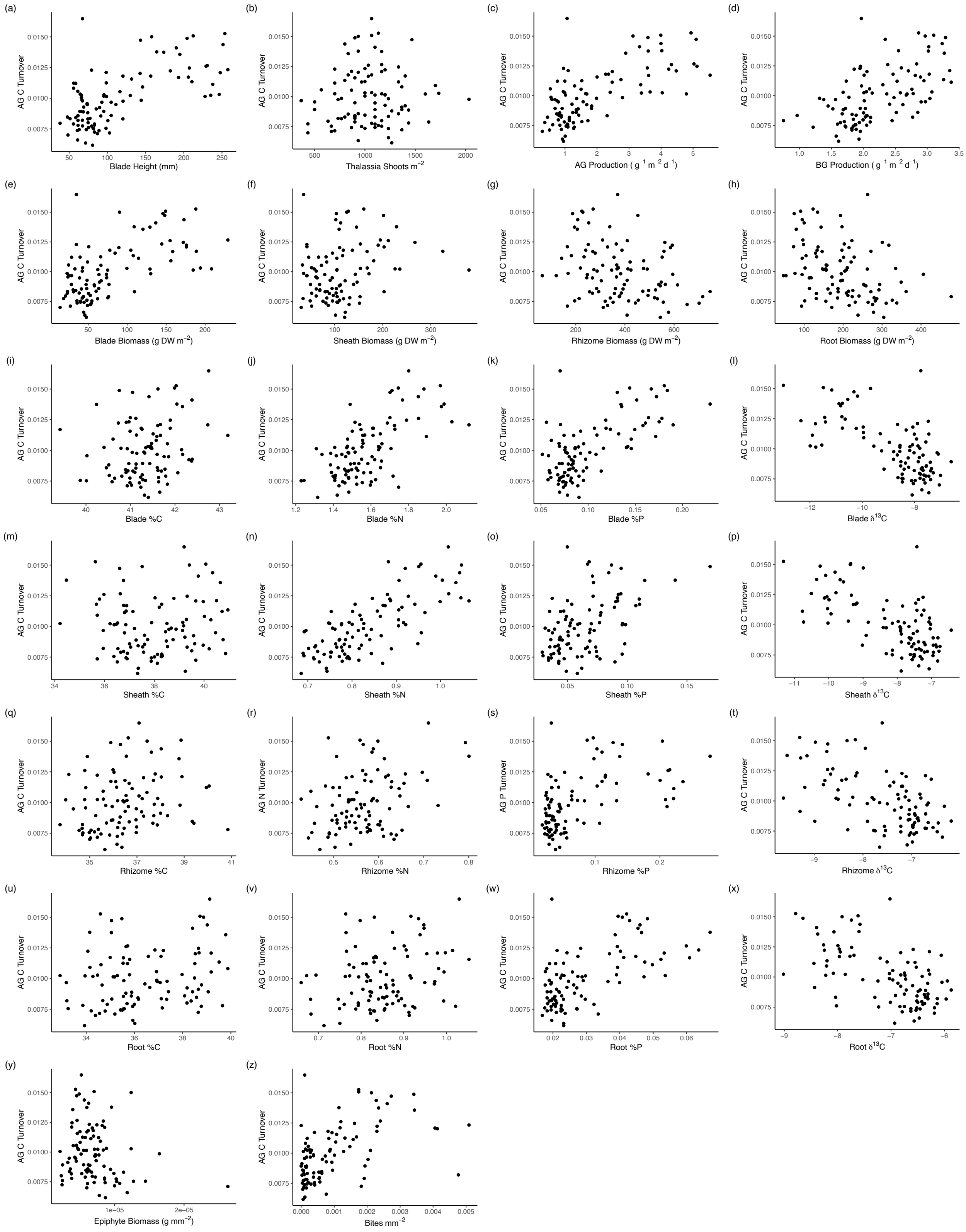

Figure S6. Correlation plots for aboveground (AG) carbon (C) turnover with all seagrass traits.

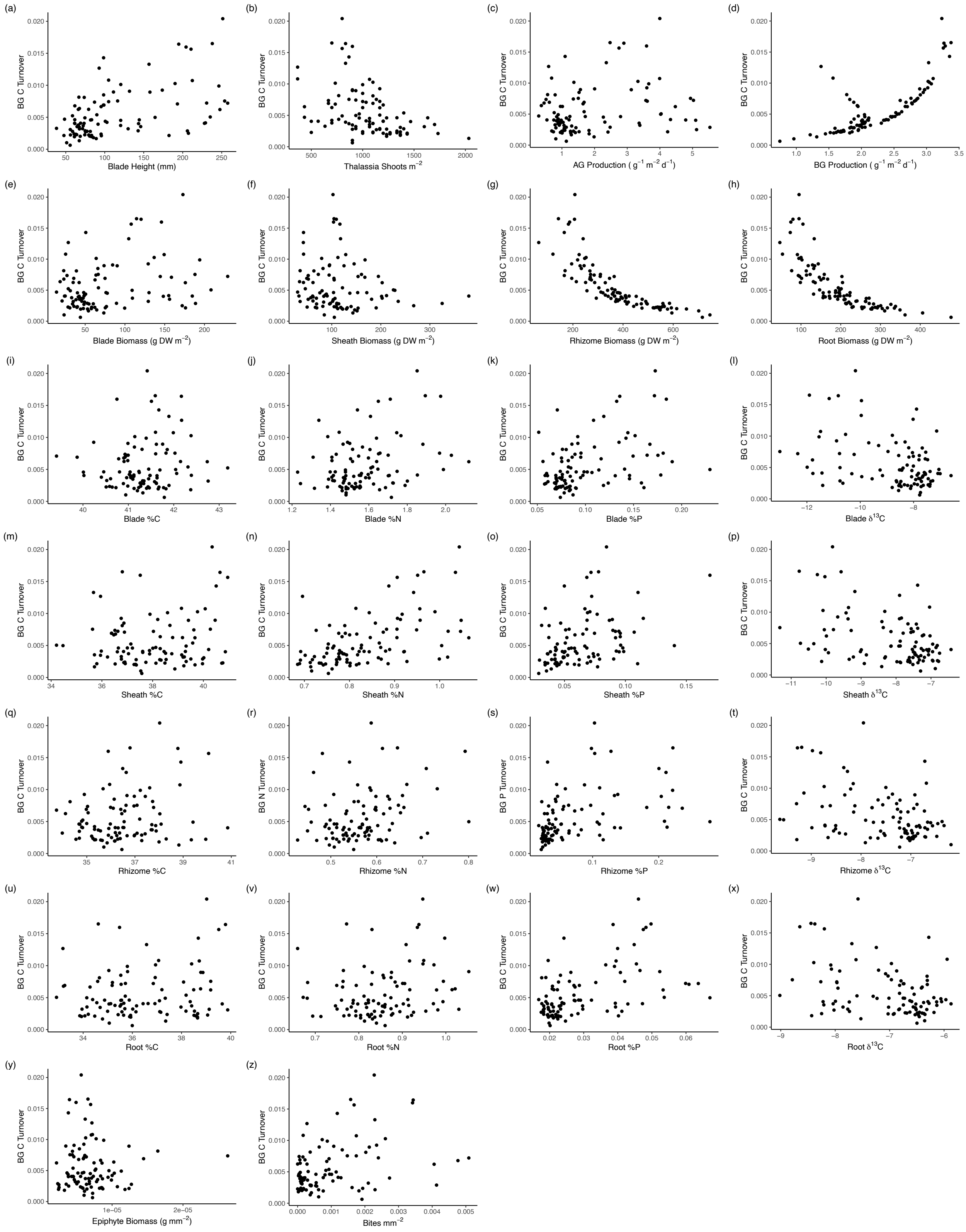

Figure S7. Correlation plots for belowground (BG) carbon (C) turnover with all seagrass traits.

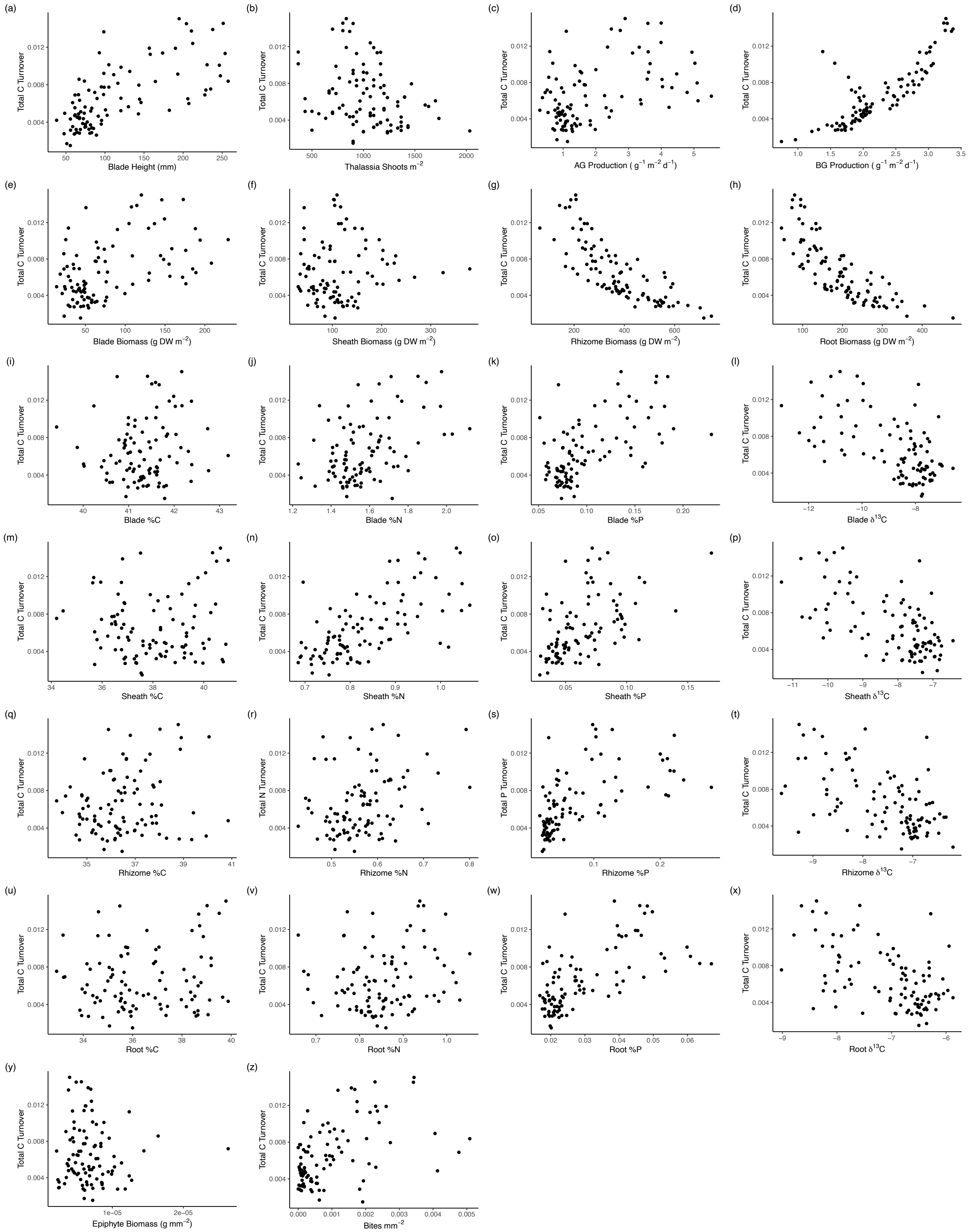

Figure S8. Correlation plots for total carbon (C) turnover with all seagrass traits.

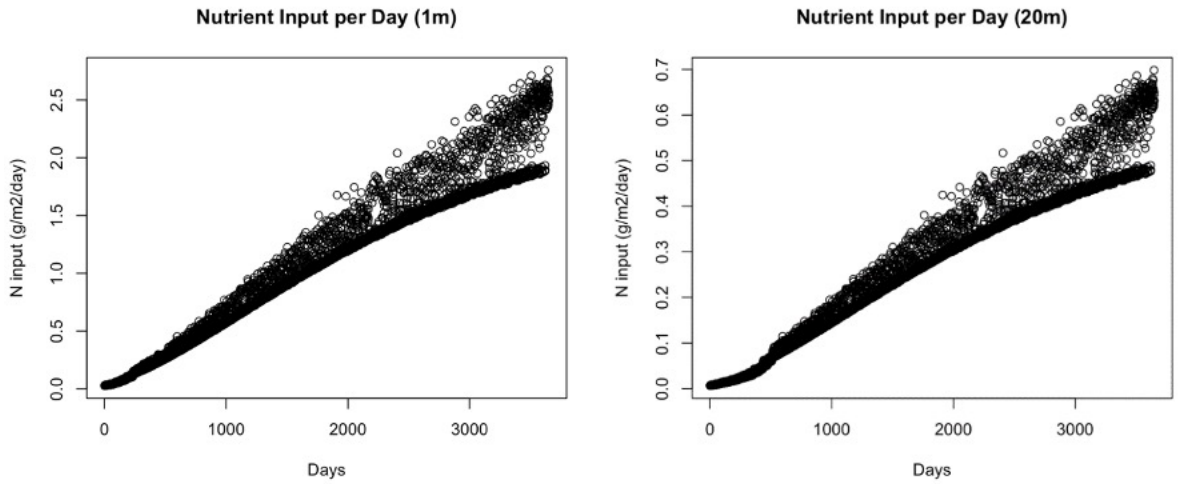

Figure S9. Nutrient input over time in model for belowground production calculations.

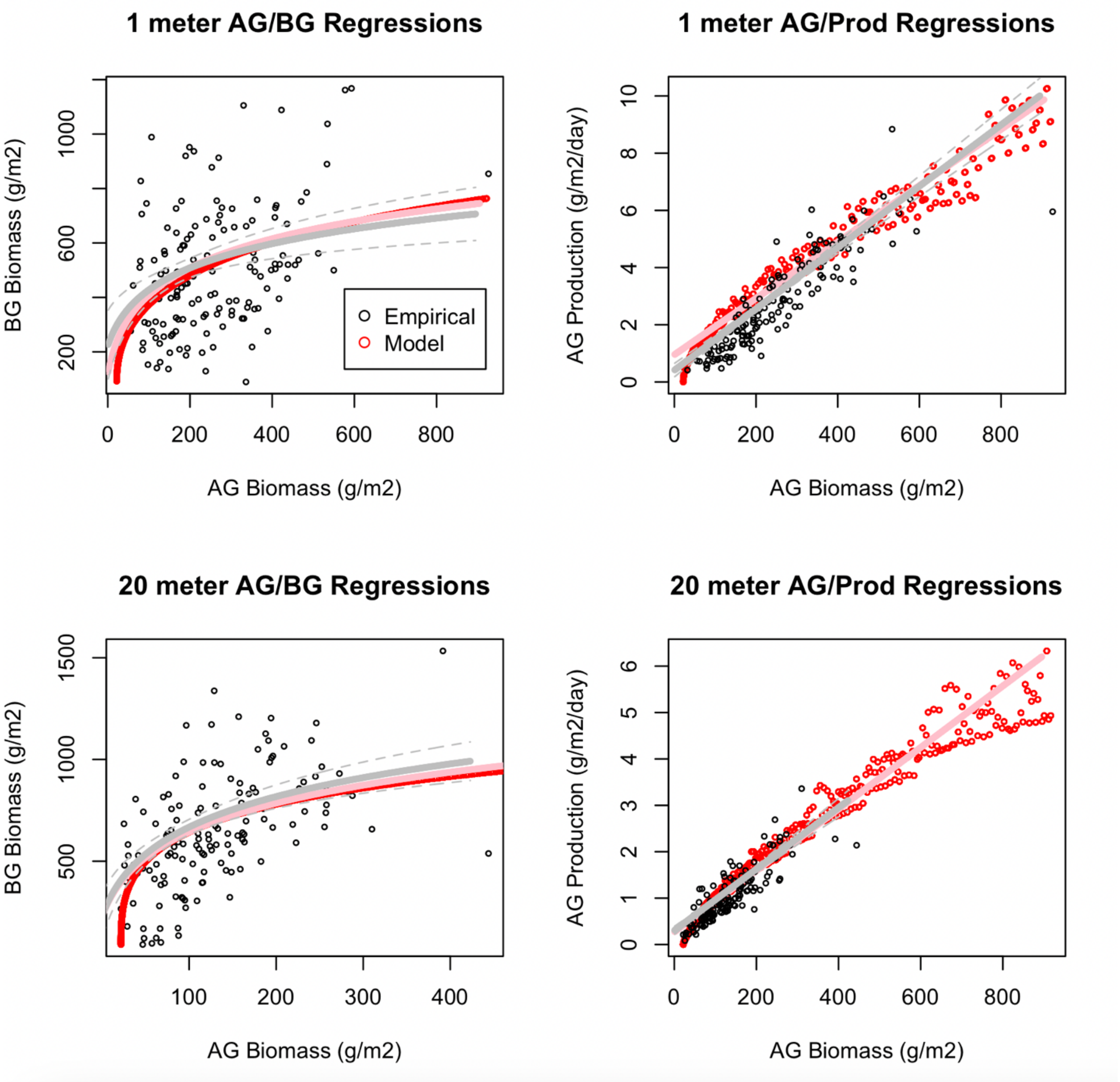

Figure S10. Comparisons between empirical (black) and model (red) data and regressions (gray and pink, respectively). Dashed gray line indicates 95% confidence interval of empirical data. The sudden increase in BG Biomass is not included in regressions of model data but is visualized to demonstrate the complete modeled range of empirical data.

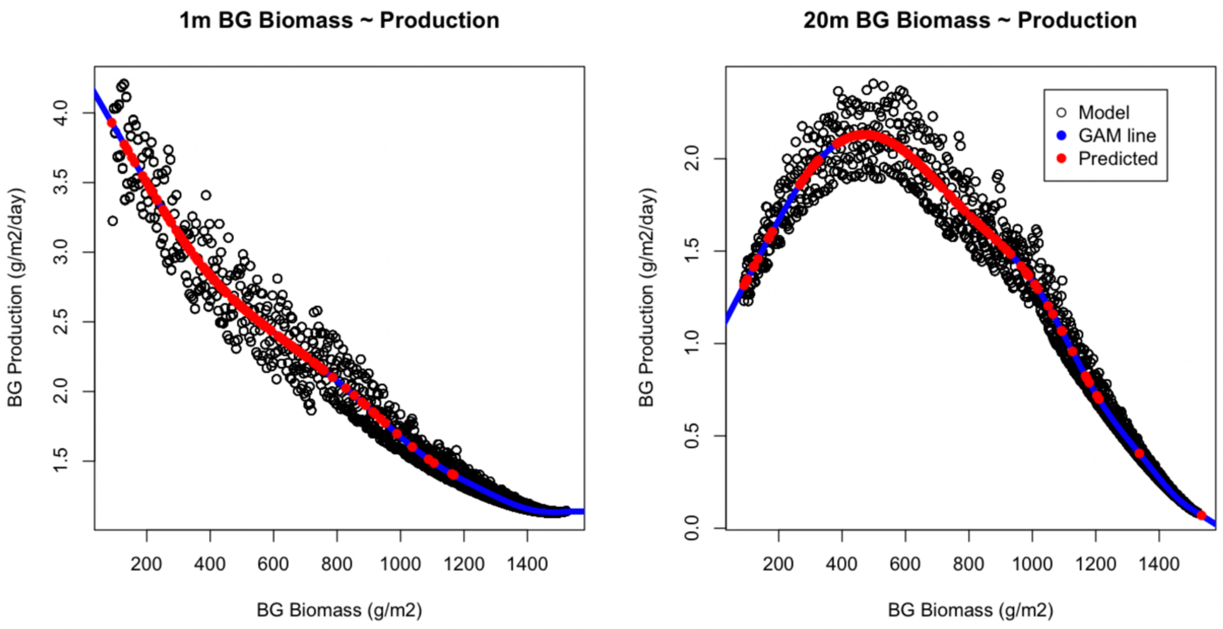

Figure S11. Belowground biomass and production relationships.
